# 3D spinal and rib cage predictors of brace effectiveness in adolescent idiopathic scoliosis

**DOI:** 10.1186/s12891-019-2754-2

**Published:** 2019-08-22

**Authors:** Saba Pasha

**Affiliations:** 1 Division of Orthopedic Surgery, The Children’s Hospital of Philadelphia, 3401 Civic Center Blvd., Philadelphia, PA 19104 USA; 2 Department of Orthopedic Surgery Perelman School of Medicine, University of Pennsylvania, Philadelphia, USA

**Keywords:** Adolescent idiopathic scoliosis, TLSO brace, Sagittal profile, Transverse plane, Predictive model

## Abstract

**Background:**

Scoliotic braces are the standard of curve for management of moderate spinal deformities in pediatric patients. The effectiveness of this treatment method has been shown; however, the spinal and rib cage parameters, in the three anatomical planes, that are associated with bracing outcome in adolescent idiopathic scoliosis (AIS) are not fully identified.

**Methods:**

A total number of 45 right thoracic AIS patients who had received a thoraco-lumbo-scaral brace for the first time were included retrospectively. For each patient, radiographic images at three visits, pre-brace, in-brace, and at least 1 year after the first brace fit were included. Age, sex, Risser sign, and curve type at pre-brace, and thoracic and lumbar frontal and sagittal Cobb angles, thoracic and lumbar apical rotations, sagittal and frontal balances at pre-brace and in-brace were determined. Two sagittal curve types (hypothoracolumbar and normal/hyperthoracolumbar kyphosis), two rib cage types based on the costovertebral joints (drooping and horizontal), and two axial shapes of the spine (S shaped and V shaped) were used to stratify the patients. Feature selection and linear regression with regularization determined the parameters and the interaction terms that predicted the brace effectiveness significantly.

**Results:**

Smaller in-brace thoracic Cobb and larger in-brace lordosis predicted brace effectiveness, *p* < 0.05. Impact of the out of brace lordosis on the brace success increased as the in brace kyphosis angle decreased, *p* = 0.046. A larger out of brace lordosis in hypothoracolumbar sagittal profile type patients improved the outcomes, *p* = 0.031. A smaller out of brace thoracic rotation improved the bracing outcomes in patients with horizontal ribs, *p* = 0.040.

**Conclusion:**

Both 3D patient specific parameters (lordosis, thoracic rotation, shape of the rib cage, and sagittal profile) and brace design (which allows larger in brace lordosis, better in brace Cobb correction) are important predictors of the brace effectiveness in AIS.

**Electronic supplementary material:**

The online version of this article (10.1186/s12891-019-2754-2) contains supplementary material, which is available to authorized users.

## Background

Scoliosis bracing is a common approach in conservative management of adolescent idiopathic scoliosis (AIS). While the etiology of AIS remains unknown, the effectiveness of bracing in controlling the curve progression has been shown [1]. Yet, scoliosis brace treatment, alone or in conjunction with other conservative treatment methods, is not always effective. Spinal curve severity changes at a varying rate as treating the patients with the brace, and in some cases progresses significantly requiring a spinal fusion surgery.

Many studies have determined the factors predicting the brace effectiveness in the AIS population. However, the findings were inconclusive [2]. The skeletal age at the time of bracing, in brace correction of the curve, initial curve severity, and compliance have been suggested as the predictors of bracing outcome [2, 3]. Brace design was also an important factor related to the curve correction [4]. While the pre-operative shape of the spine and surgical factors have predicted the surgical correction of the spine in AIS [5–7], pre-brace and brace specific parameters are not included in the bracing outcomes prediction in AIS. The impact of the spine and rib cage parameters in the three anatomical planes on management of the spinal deformity using thoraco-lumbo-scaral orthosis (TLSO) in AIS patients is not fully investigated.

The objective of this study is to identify the pre-brace and in-brace spinal curve and rib cage characteristics in the frontal, sagittal, and axial planes that predict the outcome of bracing in a cohort of right main thoracic AIS patients. It was hypothesized that both pre- and in-brace parameters of the spine and rib cage can predict the bracing outcomes in this cohort of AIS patients.

## Methods

### Subjects

**Fig. 1:**
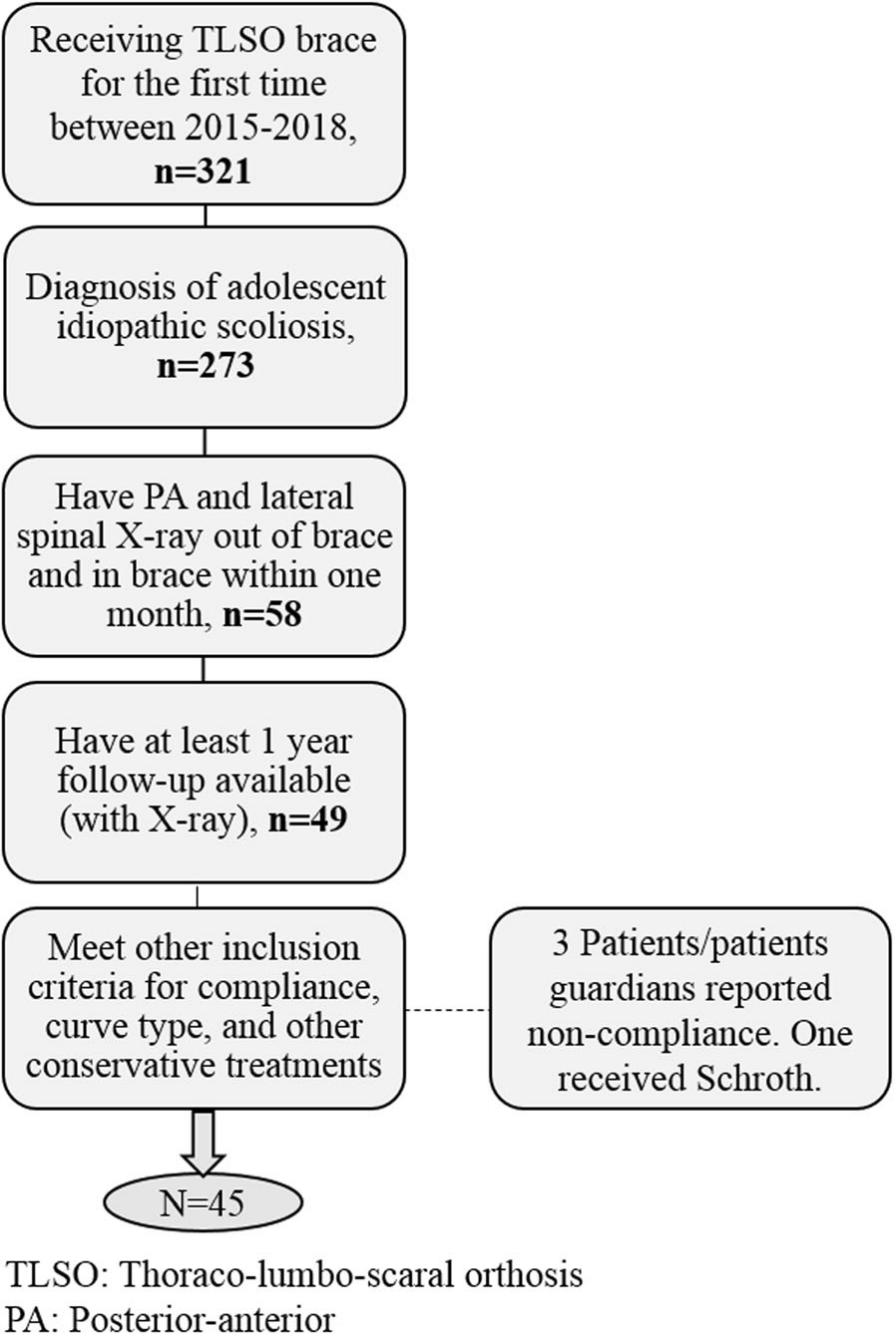
Flowchart for patient selection and inclusion criteria

The ethical committee at The Children’s Hospital of Philadelphia approved the research activities related to this study. A waiver of consent was received for this retrospective study. A total of 273 AIS patient who received a TLSO brace for the first time between 2015 and 2018 were reviewed consecutively and retrospectively. Fig. 1 shows the patient selection process. All patients had posterior-anterior and lateral spinal radiographs without brace (within 2 months prior to in-brace radiographs) and with brace (in-brace radiographs). All patients had at least one-year follow-up after the treatment was initiated (date of in brace radiographs). Patients with a minimum 16 h prescribed brace wear time were included. Only patients with an apex of the larger curve at or above T10-T11 disc were included; Patients with a main thoracolumbar curve or left thoracic curve were excluded. Patients who had prescribed any physiotherapy treatment in addition to bracing for their spinal deformity were excluded to isolate the effect of the bracing on the curve correction and eliminate the potential impact of any other therapy. A total number of 45 patients met all the inclusion criteria and were included in the study. All braces were 3D Boston brace, designed in Rodin4D software (Rodin SAS, Bordeaux, France).

### 3D reconstruction and radiographic measurements (continuous variables)

**Fig. 2:**
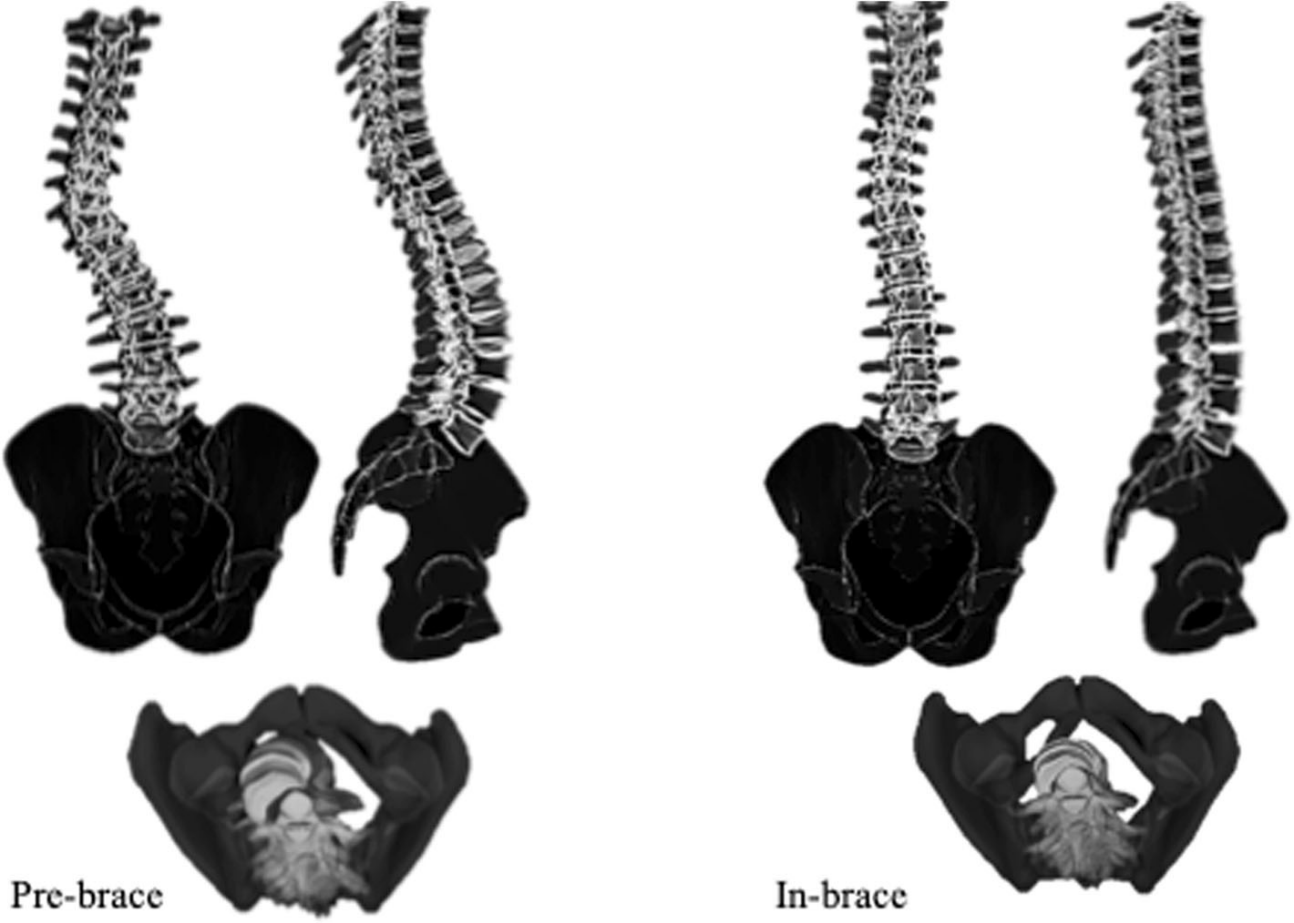
The 3D model of the spine and pelvis shown in frontal, sagittal, and axial views A) Out of brace, B) in-brace

The 3D reconstruction of the spine was generated for all the 45 patients for both pre-brace and in-brace radiographs in SterEOS 2D/3D (EOS imaging, Paris, France). Fig. 2 shows the pre- and in- brace 3D models of the spine. The 3D model was used to calculate the thoracic and lumbar Cobb angles, kyphosis (T1-T12), and lordosis (L1-S1) to avoid the error associated with 2D measurements resulted from the curve rotation or patient positioning [8, 9]. The 3D model was used to calculate the apical vertebral rotation of the thoracic and lumbar curves. Only the thoracic and lumbar Cobb angles were measured at the final follow-up because only a few of the patients had a sagittal view radiograph at one-year follow-up. Risser sign, sex, and age were noted from the patients chart at the time of in-brace radiograph.

### Patient clustering based on the 3D spinal curve types and the shape of the rib cage (binary variables)

A binary classification was used to group the patients based on their rib cage type, sagittal profile, and axial curve patterns as follows:

#### Rib cage types

**Fig. 3:**
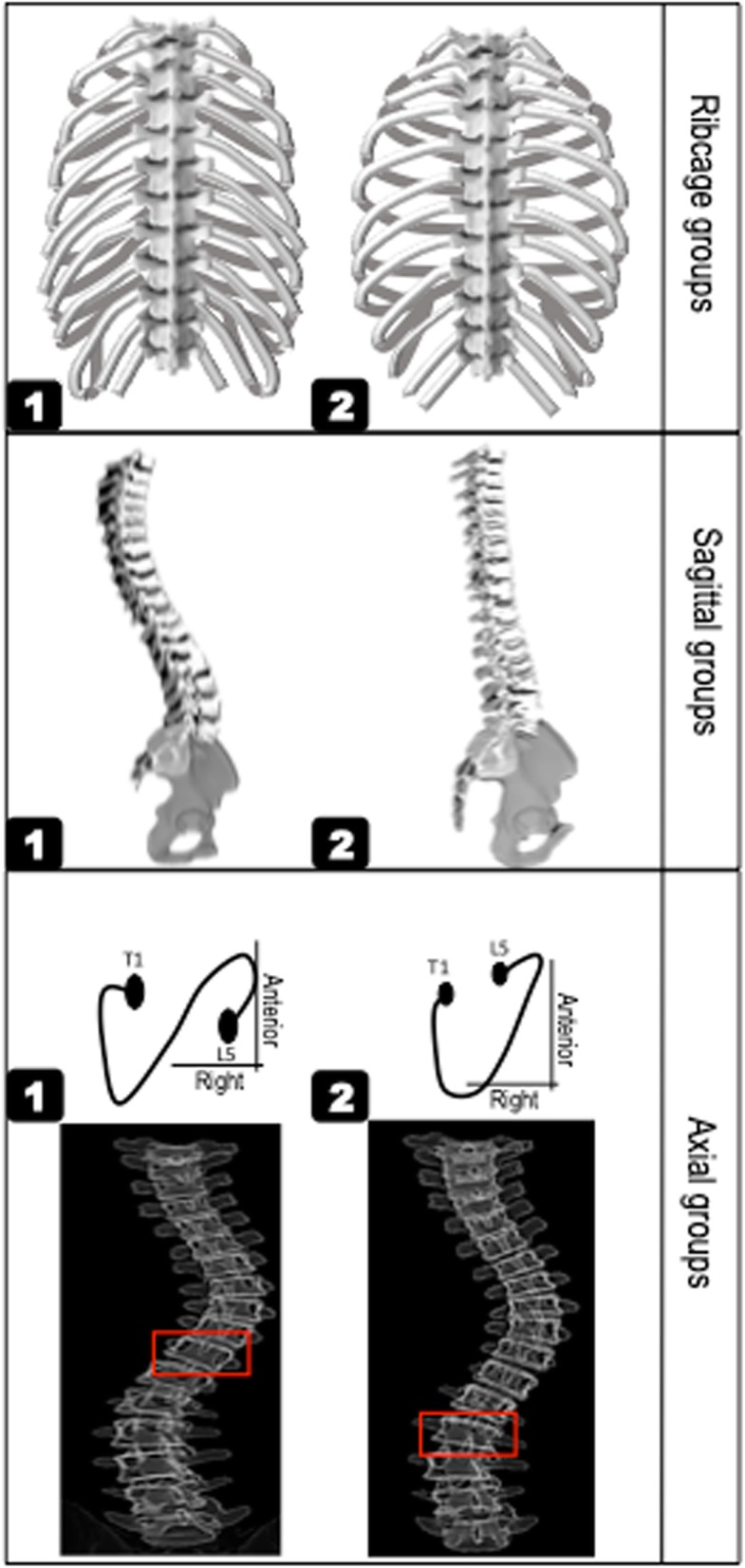
The rib cage, sagittal, and axial subtypes. Rib cage subtype included A) Type 1: asymmetric or drooping ribs, B) Type 2: horizontal ribs. Sagittal subtypes included A) Type 1: normal/hyperthrocolumnar, B) Type 2: hypothoracolumbar (flat profile). Axial types included A) Type 1: S shaped axial profile in which the direction of the vertebral rotation changes in the thoracolumbar region, B) Type2: V shaped profile with one large curve extending to the lumbar spine: the direction of the vertebral rotation changes in lower lumbar. The vertebral level at which the direction of the vertebral rotation below the apex of the thoracic curve changes is shown by red rectangles

Two rib cage types were determined using the *in-brace* radiograph: Type 1: with a rib-vertebra angle at the convex side of the apex at 60° or less (drooping ribs) [10]. Type 2: With a rib-vertebra angle at the apex more than 60° (horizontal ribs) (Fig. 3-A).

#### Sagittal profile

The *pre-brace* sagittal view determined two sagittal profile types, Type1: normal/hyperthorcolumbar types, with a kyphosis exceeding 10° and negative sagittal vertical axis indicating C7 behind the posterior aspect of the sacral endplate [6, 7]. Type 2: flat profile (hypothoracolumbar) with a kyphosis less than 10° and (Fig. 3-B).

#### Axial profile

The *pre-brace* frontal view was used to determine two axial types based on the spinal region at which the direction of the vertebral rotation changes based on the method explained in Pasha et al. [7]: Type 1- S shaped axial projection: a change in the direction of the vertebrae rotation (using the pedicle orientation) occurs in thoracolumbar spine. Type 2- V shaped axial projection: a change in the direction of the vertebrae rotation occurs at L2 or lower in lumbar spine (Fig. 3-C).

**Fig. 4:**
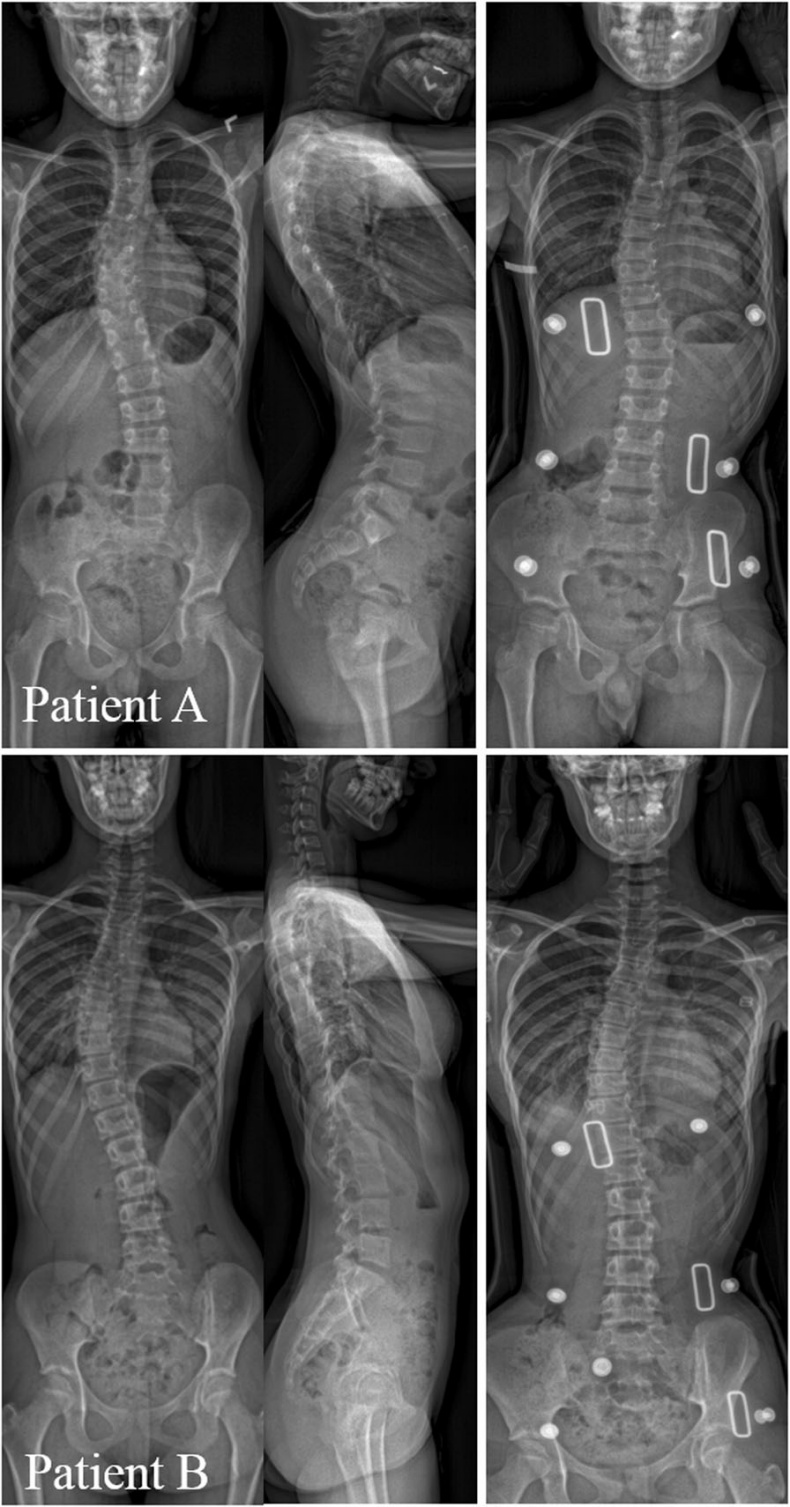
Example of patients with different rib cage, sagittal, and axial types. A) Axial Type1, Sagittal Type 1, and Rib cage Type 2. B) Axial Type2, Sagittal Type 2, and Rib cage Type1

Two examples of patients with different rib cage, sagittal, and axial curve types are shown in Fig. 4. Patient A has a rib cage Type 2 (determined from the in-brace radiograph), sagittal Type 1, and axial Type 2 (a change in the direction of vertebral rotation at L4). Patient 2 has a rib cage Type 1, sagittal Type2, and axial Type 2 (a change in the direction of vertebral rotation at L2).

### Outcome assessment

The effectiveness of the brace treatment was determined by comparing the most recent thoracic Cobb to the pre-brace thoracic Cobb angle. Brace treatment was assumed effective if the main thoracic curve had increased 5° or less (stable group). Failed treatment was assumed in case of worsening of the main Cobb angle exceeding 5° or spinal surgery for scoliosis (progressed group) [3].

### Statistical analysis

The summary statistics for the continuous and binary variables were calculated and parameters were statistically compared between the stable and progressed groups. Continuous data were compared using a T-test or Mann-Whitney U-test and the categorical data were compared using a Chi-squared test.

Finally, a predictive model using all the continuous and binary data at pre- and in-brace (Tables 1-3) and the two-level interactions between these parameters were developed; a total of 276 predictors were included in the model. To determine the most important predictors of the bracing outcomes (stable or progressed groups), a regression model with regularization and cross validation was used (Additional file 1). This method is shown to be more robust than linear regression for large number of predictors and avoids overfitting [11–13]. The predictive model was developed on 80% of the data (training dataset) and the model’s accuracy was reported on the remaining 20% of the data (test dataset).

**Table 1:** Patients’ demographics and curve characteristics at in-brace visit. Except for curve types, which was determined at the pre-brace visit, all parameters are reported at in-brace visit

Subgroups	Sex	Age (Year)	Risser sign	Curve type
Stable, *n* = 31	82% (*n* = 26) Female, 18% (*n* = 6) Male	11.3 ± 1.2	2.1 ± 1.3	54% (*n* = 17) MT, 46% (*n* = 14) RTLL
Progressed, *n* = 14	85% (*n* = 11) Female, 15% (*n* = 2) Male	12.0 ± 1.5	1.7 ± 1.2	71% (*n* = 10) MT, 29% (*n* = 4) RTLL

**Table 2:** Frontal and sagittal spinal parameters at pre-brace, in-brace and final visits

Subgroups	Visit	Thoracic Cobb (°)	Lumbar Cobb (°)	Thoracic AVR (°)	Lumbar AVR (°)	Kyphosis (°)	Lordosis (°)	Frontal balance (mm)	Sagittal balance (mm)
Stable, *n* = 31	Pre-Brace	27.2 ± 9.0	14.5 ± 10.3	−7.3 ± 5.1	9.4 ± 3.5	22.7 ± 12.3	51.6 ± 9.2	1.46 ± 1.4	−5.9 ± 9.9
	In-Brace	10.7 ± 8.4	6.0 ± 9.6	−0.6 ± 3.7	4.1 ± 0.9	17.8 ± 8.8	47.3 ± 6.3	0.54 ± 1.3	0.6 ± 6.3
	Final	26.4 ± 7.6	17.0 ± 8.9	–	–	–	–	1.32 ± 1.8	–
Progressed, *n* = 14	Pre-Brace	24.9 ± 10.5	8.1 ± 11.3	−6.7 ± 3.3	5.1 ± 4.8	19 ± 11.1	50.8 ± 16.3	2.06 ± 1.1	−3.4 ± 12.8
	In-Brace	14.2 ± 9.7	4.8 ± 11.5	−2.4 ± 2.3	1.5 ± 3.2	17.1 ± 5.4	39.9 ± 7.3	0.99 ± 1.0	−2.8 ± 8.8
	Final	39.2 ± 14.3	23.5 ± 11.4	–	–	–	–	1.08 ± 2.3	–

**Table 3:** Number of patients in sagittal, axial, and rib cage type groups in stable and progressed subgroups. The odds ratios of progression in types 1 compared to types 2 and the 95% confidence intervals are shown

	Type	Stable, n = 31	Progressed, n = 14	Odds ratios
Sagittal Type	1 2	20 11	6 8	0.41, 95% CI [0.11–1.50]
Axial Type	1 2	18 13	9 5	1.4, 95% CI [0.35–4.80]
Rib cage Type	1 2	17 14	5 9	0.46, 95% CI [0.12–1.68]

## Results

### Patients’ characteristics

A total number of 8 male and 37 female were included. The average age at the time of bracing was 11.6 ± 2.0. Patients’ sex, age, Risser sign, and curve types (single or double) are listed in Table 1. No statistically significant difference was observed between the stable and progressed groups for these parameter, *p* > 0.05 (Table 1). The average Riser sign at the final visit was 4.2 ± 0.8 [3.8–5] and 3 patients received a spinal fusion surgery.

### Radiographic measurements

Table 2 summarized the radiographic measurements in the frontal, sagittal, and axial planes at pre-brace, in-brace, and only the frontal parameters at the final visits. In-brace lordosis and final thoracic Cobb angles were significantly different between the stable and progressed groups, *p* < 0.05.

The number of patients in each rib cage, sagittal, and axial types in the stable and progressed groups and the odds ratios of progression in types 1 compared to types 2 and the 95% confidence intervals are reported in Table 3. No statistically significant differences was observed between the two groups for these variables, *p* > 0.05.

Predictive model: the methods for regression analysis with regularization and post selection [11] of significant variables are detailed in the Additional file 1. All variables in Tables 1-3 (direct effect) and their two-level interactions were included in the predictive model: a total of 276 predictors.

Least absolute shrinkage and selection operator (LASSO) identified a total of 13 of these variables to develop the predictive model while shrunk the coefficient of other variables to zero (Additional file 1). The multiple R-squared (R^2^) of the regression model, developed by these 13 variables, was R^2^ = 48.2 and the area under the receiver operating characteristic (ROC) curve was 0.69, 95% CI [0.63–0.79].

A total of 5 out of these 13 variables significantly predicted the bracing outcomes, *p* < 0.05. These variables are listed in Table 4. A smaller in-brace thoracic Cobb and larger in-brace lordosis directly associated with improved outcomes, *p* = 0.025, *p* = 0.027, respectively. For the interaction variables, the impact of pre-brace lordosis on the bracing success increased as the in-brace kyphosis angle decreased, *p* = 0.046. A larger pre-brace lordosis in sagittal Type 2 was associated with improved outcomes, *p* = 0.031. A smaller pre-brace thoracic apical vertebral rotation was associated with improved outcomes in patients with rib cage Type 2, *p* = 0.040.

**Table 4:** the predictors of the bracing outcome determined by a regression model with regularization. For the interaction terms, a positive coefficient shows an increase in the one variable also increased the effect of other variable on the outcome (Progressed group) whereas a negative coefficient suggest that an increase in a variable decreased the effect of other variable on the outcome. Coefficients shown are partial regression coefficients

	Predictor variables	Coefficients	*P* value
1	Thoracic Cobb angle (In-brace)	2.27	0.025
2	Lordosis (In-brace)	−1.93	0.027
3	Lordosis (Pre-brace) & Kyphosis (In-brace)	0.07	0.046
4	Lordosis (Pre-brace) & Sagittal Type 2	−0.99	0.031
5	Thoracic AVR (Pre-brace) & Rib cage Type 2	0.76	0.040

## Discussion

The role of pre-brace and in-brace shape of the spine and rib cage on the outcome of brace treatment in AIS patients was analyzed. The analysis showed a total of five characteristics of the spine and rib cage can moderately predict the bracing outcome in a cohort of right thoracic AIS patients with patient reported brace wear time of at least 16 h. Smaller in-brace thoracic Cobb and larger lordosis, larger pre-brace lordosis for smaller in-brace kyphosis, larger pre-brace lordosis for sagittal Type 2 (flat profile), and a smaller pre-brace thoracic rotation in patients with rib cage Type 2 (horizontal ribs) were associated with a progression less than 5° (stable group) at the most recent follow-up.

Effectiveness of the scoliotic orthotics in controlling the curve progression has been shown [1, 14]. Yet, quantitative and standardized data on the predictors of the scoliosis bracing outcome are scares. Only 26 qualitative synthesis exists on outcome evaluation of TLSO in AIS [2]. These studies include 6 different types of orthotics which vary in correction mechanisms [15]. A lack of initial in-brace correction showed strong correlation with poor outcomes [2]. The relationships between the brace wear time, initial curve severity, and Risser sign with brace treatment success were moderate or inconclusive [2]. Predictive role of Risser sign 0 compared to Risser 1 and 2 yield to both positive [3] and negative [16] outcomes. Curve types, thoracic versus lumbar, were shown to have different risks of failure [17], but other studies did not show strong relationship between the curve types and risk of failure [2]. Our study did not show differences in the curve types and Risser sign between the stable and progressed groups (Table 1); however, as shown in Table 4, the in-brace correction of the thoracic curve was a significant predictor of the outcome (Predictor #1). Moreover, in brace lumbar lordosis (L1/S1) was a predictor of bracing outcome in our cohort (Predictor #2). Previously it was thought that a smaller lordosis can impart more correction; however, the patient specific brace design showed that patients benefit from preserving the sagittal curvature [4].

The binary classifications of the spine and rib cage (Fig. 3) were performed based on the pre-brace parameters, for axial rotation and sagittal profile of the curves, and in-brace parameters, for rib cage. The brace impacted the sagittal profile and curve rotation in a similar manner for a majority of the patients; the in-brace sagittal profile was flat and the vertebrae were derotated whereas the pre-brace shape of the spine clearly differed between the patients in the sagittal curve types and the vertebral rotation patterns thus the pre-brace shape of the spine was used for sagittal and axial classification. On the other hand, the pre-brace rib cage was asymmetric for a majority of the patients thus the in-brace shape of the rib cage, as opposed to the pre-brace shape of the rib cage, was used to classify the rib cage into two groups (Fig. 3).

Studies have shown variations in the sagittal profile after brace treatment in AIS [18]. A decrease in cervical spine is shown to last 1 year following the end of brace treatment [18, 19]. Our analysis showed relationships between the pre- and in-brace sagittal parameters and bracing outcomes (Table 4). In-brace lordosis (predictor #2), interaction between the out of brace lordosis and in brace kyphosis (predictor #3), and interaction between the out of brace lordosis and sagittal types (predictor #4) were identified as sagittal predictors of bracing outcomes. For a hypothoracolumbar sagittal profile (Type 2, Fig. 1-B), a larger out of brace lordosis was a predictor of stable curve while bracing. Considering the variation in the sagittal profiles of the scoliotic patients [7] a brace design that modifies the sagittal profile can have an important affect on the bracing outcome.

3D Boston brace design uses three-point pressure system to derotate and correct the spine. Brace applies forces on the rib prominence postero-laterally and results in derotation of the spine [20], however, the role of the shape of the rib cage, particularly the angle between the ribs and vertebrae on the bracing outcomes are not well-determined. Rib-vertebrae angle differentiated between progressive versus non-progressive pediatric scoliosis [10, 21, 22]. A higher curve progression was observed in patients with an initial rib-vertebra angle asymmetry higher than 20° or a rib-vertebra angle smaller than 68° on the convex side [10]; however, it was not discussed whether this observation could have been related to the curve severity. The angle between the rib and vertebra can impact the rib loading under an exterior force [23]. A more horizontal rib deforms more under anterior-posterior forces which in turn can reduce the transferred force along the rib axis. Our results showed higher thoracic curve rotation is a risk factor of bracing failure in patients with more horizontal ribs (predictor #5, Table 4, and Fig. 1B), meaning that in patients with horizontal ribs, a larger thoracic rotation significantly reduces the effectiveness of bracing. The rib alignment with respect to the spine can impact the components of the transferred force to the spine that can contribute to the spinal curve correction. Future studies on the biomechanics of the rib orientation and the transferred force to the spine are warranted to quantify the role of the rib morphology on the spinal correction while bracing.

The study has some limitations. The analysis mainly focused on the 3D radiographic parameters and patient satisfaction and quality of life was not included. As our inclusion criteria only included patients with acceptable brace wear time, the role of the compliance was not assessed in this analysis objectively (Table 2). While the effectiveness of the thermal sensors or patient-reported brace wear time has been questioned, a reliable method for such assessment remains to be explored. Patients were not followed up up to skeletal maturity or surgery and only the 5° progression rule [3] determined the brace success or failure, yet, a majority of the patients had Risser sign of 4 or 5 showing close to or end of growth. The curve flexibility at the onset of treatment was not included in the analysis. Finally, the differences in the position of straps and slope of the trim line and the position of the pads that can impact and maintain the in-brace correction were not included in the study [4].

## Conclusions

This study included demographics and 3D spinal and rib cage parameters and their interactions, a total of 276 variables, to identify the most important predictors of the bracing outcome in a cohort of right thoracic AIS. A brace design that reduces the in brace thoracic Cobb angle, induces more lordosis and decreases kyphosis for larger lordosis curves can improve the bracing outcome. Patient specific variables, flat sagittal profile in patients with larger out of brace lordosis is associated with success whereas large thoracic rotation in patients with horizontal ribs is associated with the treatment failure.

## Additional files

Additional file 1: Regression Model with Regularization to Predict Bracing Outcome (Binary) from main effects and the two-level interactions. (DOCX 2109 kb)

## Data Availability

The patients’ dataset are confidential and are privately held for patients confidentiality safeguard.

## Abbreviations

2D: Two-dimensional
3D: Three dimensional
AIS: Adolescent idiopathic scoliosis
AVR: Apical vertebral rotation
AVT: Apical vertebral translation
LASSO: Least absolute shrinkage and selection operator
ROC: Receiver operating characteristics
TLSO: Thoraco-lumbo-scaral orthosis
