# Supplementary material for: 3D spinal and rib cage predictors of brace effectiveness in adolescent idiopathic scoliosis
Source: BMC Musculoskelet Disord. 2019 Aug 22;20:384. doi: 10.1186/s12891-019-2754-2 (PMC6706918; doi:10.1186/s12891-019-2754-2)
Supplement: Supplementary file 1 — Regression Model with Regularization to Predict Bracing Outcome (Binary) from main effects and the two-level interactions. (DOCX 2109 kb) [file 12891_2019_2754_MOESM1_ESM.docx]

**Regression Model with Regularization to Predict Bracing Outcome (Binary) from Main Effects and the two-Level Interactions**

Step 1.

Considering the large number of the variables (276 predictors) in the model, the use of a regularization technique to identify the most important predictors was explored. This method penalizes the coefficients of the variables that have a smaller effect on the response. Different methods, which apply shrinkage to the coefficient, (forcing coefficients to be exact zero in the least absolute shrinkage and selection operator (LASSO) method, forcing the coefficient close to zero in Ridge method, or a combination of ridge and LASSO in elastic net) were tested to simplify the model. This regularization is done by adding a function of shrinkage value (lambda) to the loss function that minimizes the residual sum of square (Equation 1).

Equation 1 ${Loss Function}_{Ridge}=\lambda\sum_{k=1}^{n} \beta_{k}^{2}+RSS$

${Loss Function}_{LASSO}=\lambda\sum_{k=1}^{n} {|\beta|}_{k}+RSS$

To determine which regularization technique address the bias-variance tradeoff the best in this study, reutilization terms to model were added in a way that coefficient parameter can approach zero (Ridge model) or the coefficient parameters can be exact zero in LASSO, or a combination of both in elastic net. We performed a 10 fold cross validation for each of these three models and compared the mean squared error (MSE) on the test data (20% of the data that were not used in developing the predictive model). The smallest MSE determined the most desirable regularization method for the dataset. In these models the predictors are the variables listed in table 1 to table 3 and the two-level interactions (interaction between two variables). The predicted variable is the binary bracing outcome (0= stable and 1=progressed).

Figure S.1 shows the coefficient as a function of log (lambda) regularization term- and the MSE as a function of log lambda for the LASSO, Ridge, and elastic net models. Comparing the MSEs (Table S.1), the LASSO model (alpha= 1) showed the lowest error and was selected for as the predictive model.

Figure S.1- Comparison between three models with different shrinkage terms: Ridge model, alpha=0, Elastic net, alpha=0.5, and Lasso model, alpha=1. The changes in the variable coefficients (column A) and the changes in the MSE (column B) as a function of lambda for each model are shown. The numbers on the upper-axis show the number of non-zero coefficients at each lambda value.

A

B


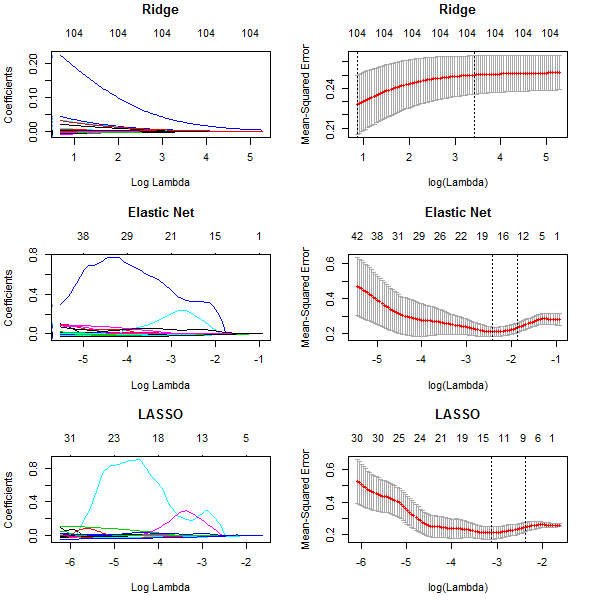


276 276

276 276

Table 1- The mean squared error (MSE) for different values of alpha

| Model | alpha | MSE |
| --- | --- | --- |
| Ridge | 0 | 0.254 |
| Elastic net | 0.1 | 0.210 |
|  | 0.2 | 0.214 |
|  | 0.3 | 0.216 |
|  | 0.4 | 0.217 |
|  | 0.5 | 0.216 |
|  | 0.6 | 0.218 |
|  | 0.7 | 0.218 |
|  | 0.8 | 0.218 |
|  | 0.9 | 0.214 |
| LASSO | 1 | 0.184 |

Step 2. After determining the model (step 1), to choose the regularization parameter a 5 fold cross validation with a LASSO model on a series of lambda values. Figure 2.S shows model MSE for the lambda values. MSE gets smaller for large values of the regularization parameter (Lambda), suggesting smaller number of variables generated a better predictive model of the training set. The Lambda value at which the MSE was minimized was selected to develop the final model and determined the penalized coefficient that more significantly predict the outcome. The variables with highest coefficients are listed in table 2.

Figure 2.S. Mean squared error calculated for LASSO model for different lambda values.

Table 2.S. shows the variables that were selected by LASSO regression to be included in the predictive model. In the manuscript we identified whether these parameters significantly predicted the results using LASSO inference post selection (non-zero coefficients in the sparse matrix).


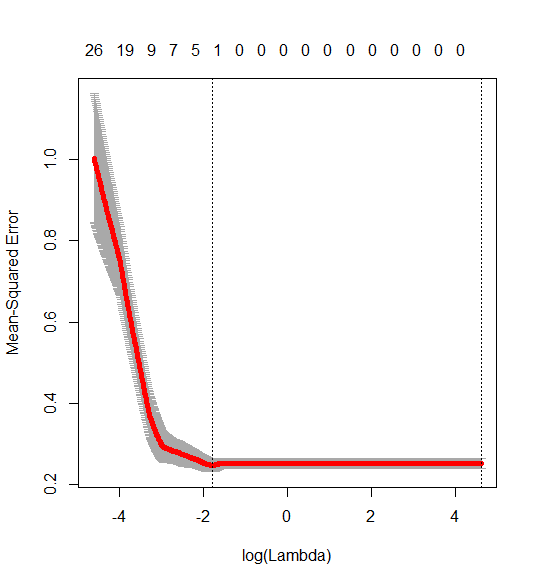


| Predictor variables | Coefficients (logit) |
| --- | --- |
| (Intercept) | 0.888130 |
| Thoracic Cobb (In) | 0.004567 |
| Lordosis (In) | -0.013684 |
| Lordosis (Out) | 0.02459 |
| Lumbar Cobb (Out) & Thoracic AVR (Out) | 0.018902 |
| Lumbar Cobb (Out) & Thoracic AVR (In) | 0.014685 |
| Lumbar Cobb (In) & Thoracic AVR (Out) | 0.0017502 |
| Lumbar Cobb (Out) & Axial Type2 | -0.066323 |
| Lumbar Cobb (In) & Axial Type2 | 0.1988396 |
| Lordosis (Out) & Kyphosis (In) | 0.001983 |
| Lordosis (Out) & Sagittal Type2 | 0.013773 |
| Thoracic AVR (Out)& Ribcage Type 2 | -0.10871373 |
| Ribcage symmetry & Axial Type 2 | 1.59394316 |
| Sagittal Type2& Ribcage Type 2 | 0.15653240 |
